# Isolation of photoprotective signal transduction mutants by systematic bioluminescence screening in ***Chlamydomonas reinhardtii***

**DOI:** 10.1038/s41598-019-39785-z

**Published:** 2019-02-26

**Authors:** Ryutaro Tokutsu, Konomi Fujimura-Kamada, Tomohito Yamasaki, Takuya Matsuo, Jun Minagawa

**Affiliations:** 1 Division of Environmental Photobiology, National Institute for Basic Biology, Nishigo-naka 38, Myodaiji, Okazaki, 444-8585 Japan; 2 Department of Basic Biology, School of Life Science, Graduate University for Advanced Studies, Okazaki, 444-8585 Japan; 3 Core Research for Evolutional Science and Technology, Japan Science and Technology Agency, Saitama, 332-0012 Japan; 4 Science and Technology Department, Natural Science Cluster, Kochi University, 2-5-1 Akebono-cho, Kochi, 780-8520 Japan; 5 Center for Gene Research, Nagoya University, Nagoya, 464-8602 Japan

## Abstract

In photosynthetic organisms, photoprotection to avoid overexcitation of photosystems is a prerequisite for survival. Green algae have evolved light-inducible photoprotective mechanisms mediated by genes such as light-harvesting complex stress-related (*LHCSR*). Studies on the light-dependent regulation of *LHCSR* expression in the green alga *Chlamydomonas reinhardtii* have revealed that photoreceptors for blue light (phototropin) and ultraviolet light perception (UVR8) play key roles in initiating photoprotective signal transduction. Although initial light perception via phototropin or UVR8 is known to result in increased *LHCSR3* and *LHCSR1* gene expression, respectively, the mechanisms of signal transduction from the input (light perception) to the output (gene expression) remain unclear. In this study, to further elucidate the signal transduction pathway of the photoprotective response of green algae, we established a systematic screening protocol for UV-inducible *LHCSR1* gene expression mutants using a bioluminescence reporter assay. Following random mutagenesis screening, we succeeded in isolating mutants deficient in *LHCSR1* gene and protein expression after UV illumination. Further characterization revealed that the obtained mutants could be separated into 3 different phenotype groups, the “UV-specific”, “*LHCSR1*-promoter/transcript-specific” and “general photoprotective” mutant groups, which provided further insight into photoprotective signal transduction in *C*. *reinhardtii*.

## Introduction

In photosynthesis, solar energy is utilized by photochemical reactions in two photosystems (PSI and PSII)^1^. PSII and PSI are connected in series and convert absorbed light energy into chemical energy via photosynthetic electron transfer. The products of photosynthetic electron transfer, NADPH and ATP, are essential substrates for carbon fixation via the Calvin–Benson–Bassham cycle. The fixed carbon source provided by this cycle is then available for sustaining plant growth. In nature, to appropriately utilize incident solar energy under various environmental conditions, photosynthetic organisms optimize their light-harvesting capacity^2^. Because excess light energy can rapidly lead to overexcitation of photosystems, which results in reactive oxygen species production within the chloroplast^3^, photoprotection against excess light is essential for photosynthetic organisms. To avoid harmful overexcitation, plants and algae have established photoprotective mechanisms that dissipate excess light energy as thermal energy in a process called non-photochemical quenching (NPQ)^4^.

Several key components for NPQ activation have been discovered among photosynthetic organisms^5^. PsbS was irst discovered in land plants as a subunit of PSII and was recognized as an essential factor for rapid NPQ activation^6^. The PsbS protein in green algae also contributes to NPQ and is associated with PSII, similar to the PsbS in land plants^7,8^. Light-harvesting complex stress-related (LHCSR) proteins have also been reported as important NPQ factors in both moss and green algae^9,10^. Mutants deficient in either LHCSR1 or LHCSR3 expression demonstrate a severe high-light (HL)-sensitive phenotype^9,11^, suggesting that these proteins are required for the survival of *Chlamydomonas reinhardtii* under excess-light conditions. Recent studies have clarified that LHCSR3 associates with PSII and substantially contributes to excitation energy dissipation in PSII supercomplexes^12^. LHCSR1, not only thermally dissipates excitation energy on the light-harvesting complex II (LHCII), but also mediates it from LHCII to PSI at low pH, thus allowing green algae to alleviate overexcitation of PSII at the cost of PSI excitation^13^. Collectively, these NPQ-related proteins play crucial roles in photoprotection in both land plants and green algae.

In sharp contrast to the constitutive PsbS protein expression present in land plants, the photoprotective proteins in green algae such as PSBS, LHCSR1 and LHCSR3 are light inducible. We recently demonstrated that *LHCSR3* gene expression is mediated by blue light perception via the photoreceptor phototropin^14^. Although the other NPQ component, *LHCSR1*, is an HL-inducible gene^9^, the exact cues triggering the expression of this photoprotective gene was unknown until recently. Allorent *et al*. reported that *LHCSR1* and *PSBS* gene expression is mainly under the control of ultraviolet (UV) signal transduction, which is initiated by UV perception via the photoreceptor UVR8^11^. The deficiency in UVR8 resulted in severe chlorophyll bleaching under HL^11^, suggesting that the presence of LHCSR1 and/or PSBS effectively protect PSII under light with UV components because UV light is very detrimental to the oxygen-evolving manganese cluster of PSII^15^. Thus, the light-inducible activation of photoprotection via the photoreceptors in green algae is crucial to avoid photodamage^16^.

However, signal transduction pathways following light illumination remain largely unclear. To elucidate the signal transduction pathways in *C*. *reinhardtii*, we established a systematic forward mutagenesis screening protocol based on a luciferase-dependent bioluminescence reporter assay. In this system, we fused a firefly luciferase to the *LHCSR1* gene (including its 5′ UTR sequences) for monitoring UV-dependent activation of *LHCSR1* expression as described in a previous study^17^. Here, we isolated and characterized mutants deficient in *LHCSR*-dependent photoprotection (*DSR*). Intriguingly, among the *DSR* mutants isolated, three displayed a severe chlorophyll bleaching phenotype under HL conditions, implying that the mutants were deficient in not only UV-inducible photoprotection but also overall photoprotection.

## Results and Discussion

**Figure 1:**
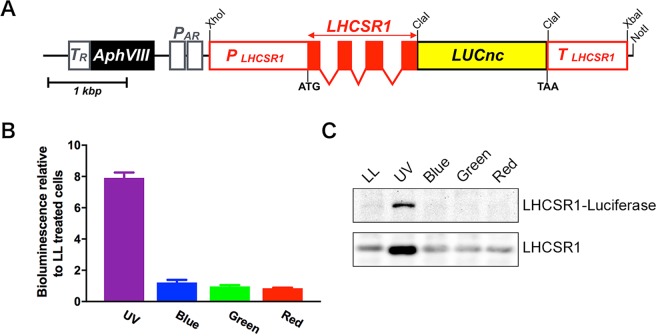
Construction and characterization of the *LHCSR1-luciferase* reporter strain. (**A**) Construction map for introducing the *LHCSR1-luciferase* reporter gene into *Chlamydomonas* cells. *P*_*AR*_ and *P*_*LHCSR1*_ indicate the *HSP70A-RBCS2* tandem promoter and *LHCSR1* promoter regions, respectively. *T*_*R*_ and *T*_*LHCSR1*_ indicate the terminator regions of *RBCS2* and *LHCSR1*, respectively. The *LHCSR1* gene, including exons and introns, is represented by red boxes. The codon-optimized firefly luciferase (*LUCnc*) gene and paromomycin resistance gene (*AphVIII*) are represented by the yellow box and black box, respectively. (**B**) Bioluminescence activity of the reporter strain under various colored lights. The intensities of the fluorescent light with UV and the blue (450 nm), green (530 nm) and red (660 nm) monochromatic LED lights were 30 μmol/m^2^/s. Bioluminescence activities of the light treated cells were normalized to the bioluminescence activity under LL. Data represent the mean ± SEM of three biological replicates. Only UV-treated cells showed a significant increase in activity (one-way ANOVA, *P* < 0.0001). (**C**) Immunoblot analysis of LHCSR1 and LHCSR1-luciferase fusion protein in the reporter strain obtained in (**B**) using a specific antibody against LHCSR1. The signals were obtained from the same blot at different exposure times (short and long exposure for LHCSR1 and LHCSR1-luciferase signals, respectively). Raw images of the blot are shown in the supplemental information (Supplementary Fig. S2). The samples illustrated here are representative of three biological replicates.

To establish the systematic random mutagenesis-based screening of UV-inducible photoprotection mutants, we first attempted to construct a reporter system using promoter regions of UV-inducible genes in *C*. *reinhardtii*. Since the LHCSR1 protein had previously been investigated as a UV-inducible photoprotective factor^11^, we decided to use the *LHCSR1* gene with its promoter region (i.e., the region 1.2 kb upstream). The selected sequence was then fused with firefly luciferase cDNA (*LUCnc*)^17^ to generate a LHCSR1-luciferase translational fusion reporter gene (Fig. 1A; also see Methods). A plasmid including the *LHCSR1-LUCnc* gene was introduced into the nuclear genome of the wild-type *C*. *reinhardtii* strain (137c mt+ ), and transformants were selected on tris-acetate-phosphate (TAP^18^) agar plates containing paromomycin.

To generate a strain expressing sufficient levels of both LHCSR1 and LHCSR1-luciferase fusion proteins for subsequent random mutagenesis screening, we first screened ~1,000 LHCSR1-luciferase transformants after 4 hours of UV irradiation. As a result, we observed obvious UV-inducible bioluminescence in 56 transformants. After testing whether the UV-inducible bioluminescence activity in the transformants was reproducible or not, we successfully isolated 8 transformants showing significant bioluminescence activity after UV illumination (Supplementary Fig. S1). We also briefly checked the LHCSR1 and LHCSR1-luciferase fusion proteins in the top 3 bioluminescence candidates of the reporter strain (#443, #479, and #717). Both authentic LHCSR1 and LHCSR1-luciferase fusion proteins clearly accumulated in these strains after UV illumination (Supplementary Fig. S2). When these results were considered with the UV-inducible bioluminescence in these candidate reporter strains, it was plausible that the LHCSR1-luciferase fusion protein-dependent bioluminescence activity in these reporter strains roughly correlated with authentic LHCSR1 protein accumulation. For the following bioluminescence-based screening, we decided to use an *LHCSR1-Luc717* strain as a suitable recipient strain because the strain showed the highest bioluminescence activity among the candidates. This *LHCSR1-Luc717* strain showed LHCSR1 and LHCSR3 protein expression almost comparable to the wild-type (WT) cells under UV light and blue light, respectively (Supplementary Fig. S3). The LHCSR1-luciferase fusion protein was also detected in the *LHCSR1-Luc717* strain, indicating that the reporter gene was successfully introduced to and expressed in *C*. *reinhardtii*. Accordingly, the *LHCSR1-Luc717* strain showed slightly increased NPQ capacity, particularly after UV treatment (Supplementary Fig. S4), suggesting that the LHCSR1-luciferase fusion protein may function in photoprotection in the reporter strain. To evaluate the UV specificity of the reporter system, we next measured bioluminescence and LHCSR1-luciferase protein expression in the *LHCSR1-Luc717* strain. When cells were treated with UV, a significantly increased luciferase activity (~8-fold) was detected, while other light treatments resulted in a less than 1.5-fold increase in its activity (Fig. 1B). Accompanying the increased luciferase activity induced by UV, the LHCSR1-luciferase fusion protein accumulated under only UV conditions (Fig. 1C). These results strongly indicate that the UV-induced increase in bioluminescence of the *LHCSR1-Luc717* strain resulted from activation of the *LHCSR1* promoter and/or possibly stabilization of *LHCSR1* mRNA, which in turn increased the levels of LHCSR1-luciferase fusion protein. We concluded that this strain of *LHCSR1-Luc717* was suitable for further luciferase activity-based mutant screening.

**Figure 2:**
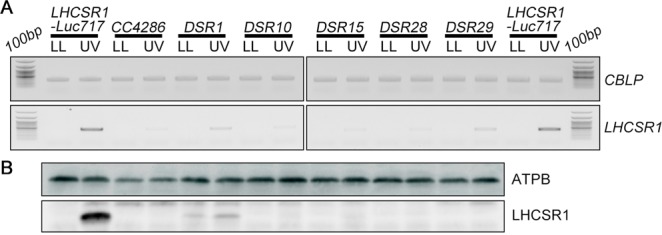
*LHCSR1* expression in isolated mutants following UV induction. (**A**) Gene expression analysis of *LHCSR1* by RT-PCR. The *CBLP* gene was used as the housekeeping control. RNA and protein samples were collected after 1 hour of irradiation with UV. A 100-bp DNA ladder is shown. (**B**) Expression of the LHCSR1 protein was detected using specific antibodies, and the ATPB protein was used as the loading control. The protein samples were collected after 4 hours of irradiation with UV. Samples illustrated here are representative of three biological replicates.

We next generated an insertional mutant library with an *aph7* gene conferring hygromycin resistance^19^ to isolate UV-inducible photoprotection mutants. Use of this approach allowed ~23,500 transformants to be isolated by selection on TAP medium containing hygromycin. To further select for the UV-inducible photoprotection mutants among the transformants, luciferase activity was measured with bioluminescence monitoring after UV treatment. Five mutants (*DSR1*, *10*, *15*, *28*, and *29*) that were deficient in both *LHCSR1* gene expression (Fig. 2A and Supplementary Fig. S5) and LHCSR1 protein accumulation (Fig. 2B) were obtained. All these mutants had no differences in the *LHCSR1* gene sequence, including the region used for the construct shown in Fig. 1, implying a mutation in the upstream signal transduction that activates the UV-inducible *LHCSR1* promoter and/or a mutation in the factor that stabilizes *LHCSR1* mRNA in these mutants.

**Figure 3:**
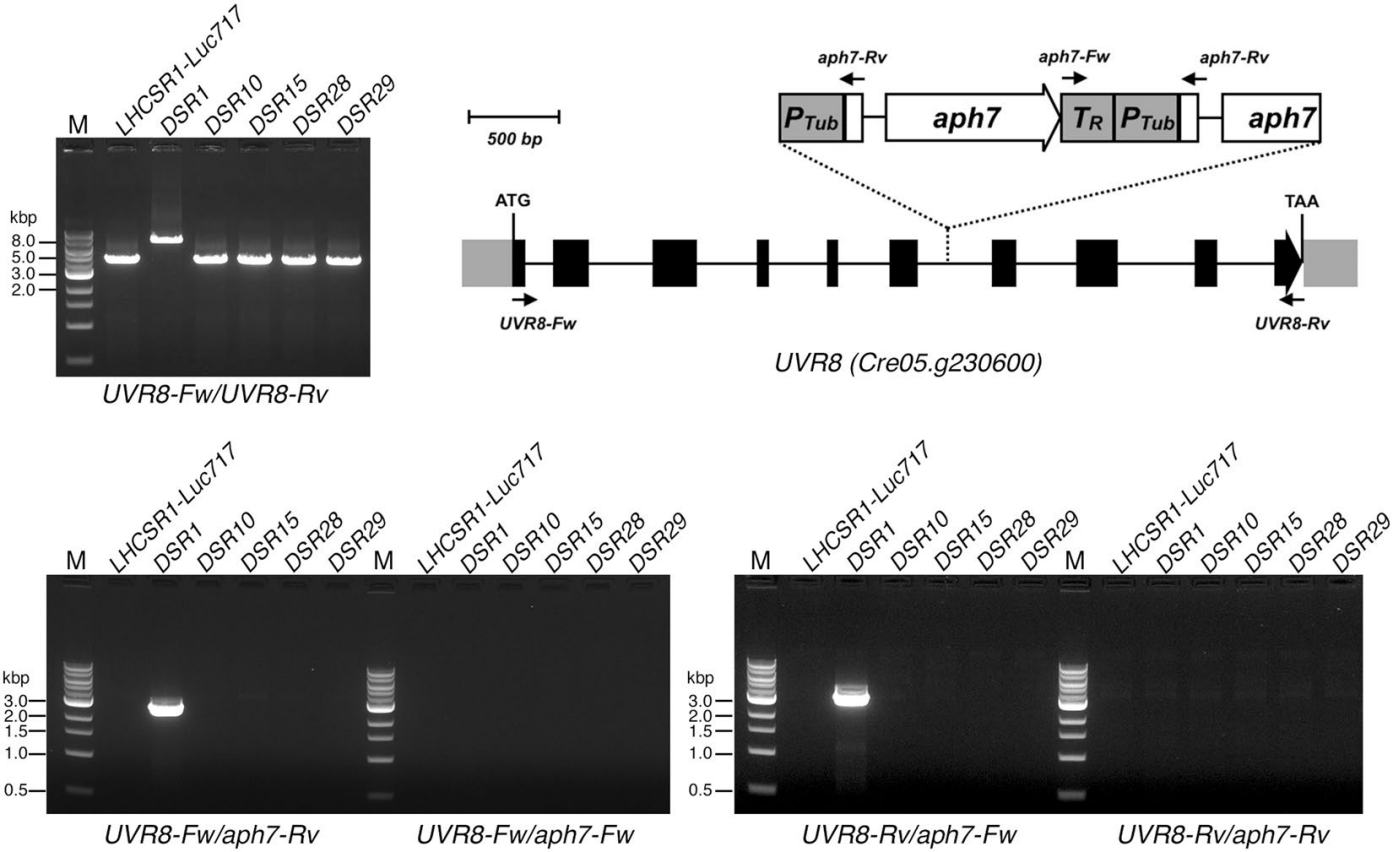
Genomic PCR of the *UVR8* gene in the *DSR* mutants. Genomic PCR analysis using primers for the *UVR8* gene and the *aph7* cassette. The primer combinations used are shown under the gel pictures. M, 1-kb DNA ladder. A schematic diagram of the gene structure (chromosome) of *UVR8* (*Cre05*. *g230600*) is illustrated at the upper right. The translation start and stop codons and the position and orientation of the inserted a*ph7* tags in the *DSR1* mutant identified in this study are all indicated.

It has been reported that the UVR8 protein is responsible for UV perception-based initiation of the photoprotective signal transduction pathways in green algae^11^. To determine whether the *aph7* cassette in the *DSR* mutants was inserted into the *UVR8* gene, we further evaluated the mutants by genomic PCR. Genomic PCR with primers complementary to either the *UVR8* gene or the *aph7* cassette produced PCR products for both only in the case of *DSR1* (Fig. 3). The exact insertion sites were confirmed to be in the sixth intron of the *UVR8* gene by sequence analysis of the amplicons (Fig. 3, upper right scheme). We thus confirmed the previous report that UVR8 is an essential signal transduction factor in UV-inducible photoprotection in *C*. *reinhardtii*^11^. These data additionally led us to conclude the successful systematic screening of UV-inducible photoprotection mutants in this study.

Since the *LHCSR1-Luc717* strains showed a clear phenotype where the expression of both the *LHCSR1* gene and protein were strongly correlated with luciferase bioluminescence activity, we performed linkage analysis of the mutants to clarify whether the observed mutant phenotypes were induced by a single-locus insertion of an *aph7* cassette. Prior to linkage analysis, generation of the opposite mating-type strain (mt−) equivalent to the *LHCSR1-Luc717*(mt+ ) strain was required. To generate the *LHCSR1-Luc717*(mt−) strain, we crossed the *LHCSR1-Luc717*(mt+ ) strain with the *CC4286*(mt−) strain as described in the Methods section because whole-genome sequencing of the *CC4286* strain showed that it has the highest similarity to the WT strain (*137c*, mt+ ) used in this study^20^. In this process, we found that the *CC4286* strain had a mutation that caused a defect in UV-inducible LHCSR1 expression (Fig. 2). We next used linkage analysis to validate whether the mutants harbor a single-locus *aph7* insertion. All mutants were backcrossed with the *LHCSR1-Luc717*(mt−) strain, and tetrads were dissected. The resultant progenies were subjected to bioluminescence measurements and tested for hygromycin resistance. Where the mutant phenotype was caused by a single integration of the marker gene into the genome, the phenotype was segregated 2:2 and completely cosegregated with hygromycin resistance in linkage analyses. Since *DSR1*, *DSR10* and *DSR15* showed complete cosegregation of the mutant phenotype and hygromycin resistance in all dissected tetrads (Supplementary Table S1), we concluded that these mutants have a single-locus functional *aph7* cassette insertion responsible for the defect in UV-inducible *LHCSR1* expression. On the other hand, some of the mutants (*DSR28* and *DSR29*) showed incomplete cosegregation between hygromycin resistance and the mutant phenotype, implying that insertion of the functional *aph7* cassette was not responsible for their phenotype.

**Figure 4:**
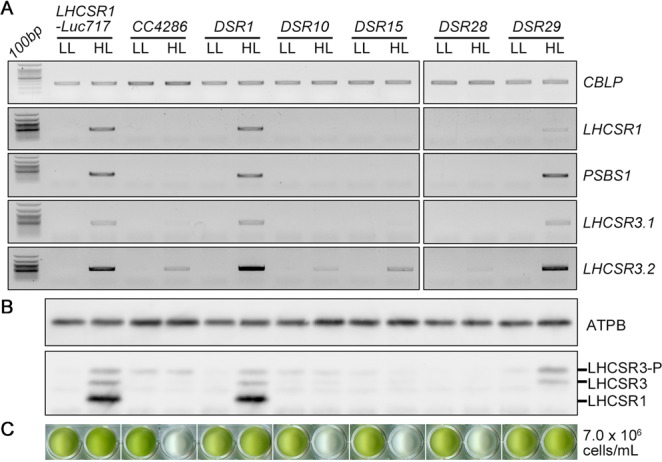
Photoprotective gene and protein expression in the isolated mutants under high-light conditions, including UV. (**A**) Genes related to photoprotection, *LHCSR1*, *LHCSR3* and *PSBS1*, were analyzed with RT-PCR. The *CBLP* gene was used as the housekeeping control. A 100-bp DNA ladder is shown. Gel images for each gene were cropped from the same gel. (**B**) The LHCSR1 and LHCSR3 proteins were detected using specific antibodies against each. The ATPB protein was used as the loading control. LHCSR3-P represents LHCSR3-phosphorylated. (**C**) The bleaching phenotype of the mutants was visualized in a multiwell plate. The concentration was normalized to 7.0 × 10^6^ cells/mL. Both the RNA and protein samples were collected after 1 hour, and the cultures were collected after 8 hours of irradiation under high-light with UV conditions at 300 μmol/m^2^/s. The samples illustrated here are representative of three biological replicates.

**Figure 5:**
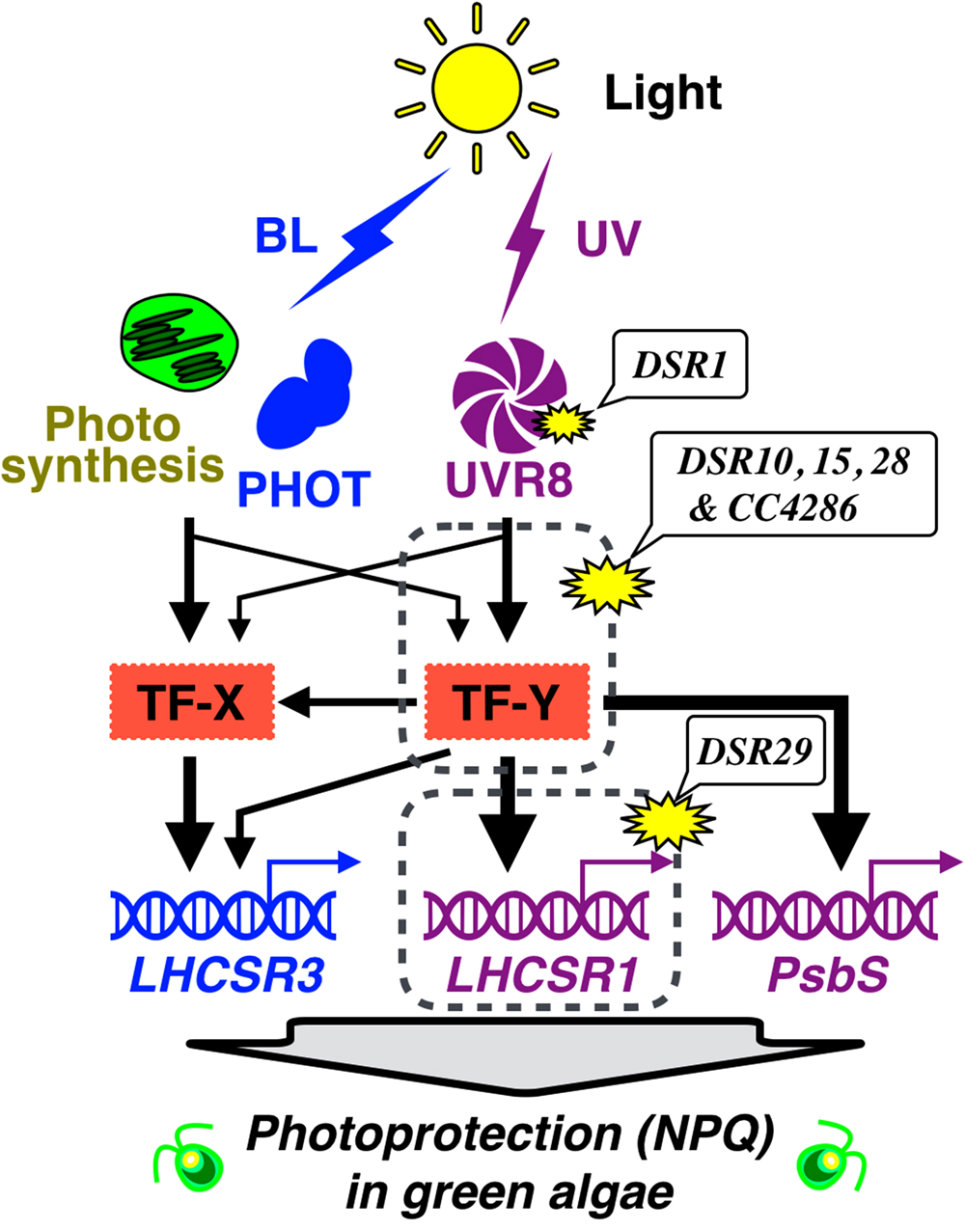
Isolated mutants in the schematic signal transduction of the photoprotective response in green algae. Light color (BL: blue light and UV: ultraviolet light)-dependent photoprotective signal transduction mediated by photosynthesis (depicted as chloroplast) and photoreceptors (PHOT: phototropin and UVR8) are shown. Two possible transcription factors (TF-X and TF-Y) are drawn as red boxes. The thickness of each arrow represents the extent of predicted signaling contributions. The predicted site (or area indicated by gray dashed boxes) of the mutants obtained in this study are indicated as yellow stars.

Finally, to explore whether these mutations affect only *LHCSR1* expression or general photoprotective gene expression, all mutant strains isolated in this study were treated with HL including UV to evaluate their photoprotective capability (Fig. 4). The *LHCSR1-Luc717* strain showed expression of all representative photoprotective genes, including *LHCSR1*, *PSBS1* and *LHCSR3*, under HL-UV conditions (Fig. 4A). Immunoblot analysis demonstrated that both LHCSR1 and LHCSR3 proteins substantially accumulated within an hour (Fig. 4B) to achieve HL tolerance (Fig. 4C). *CC4286*, *DSR10*, *DSR15* and *DSR28* showed a severe deficiency in the expression of all photoprotective genes and proteins, which in turn correlated with chlorophyll bleaching of the cells. Given that these mutants showed an almost comparable photosynthetic electron flow rate to the *LHCSR1-Luc717* strain (Supplementary Fig. S6), these results clearly indicated that these mutants were deficient in photoprotection due to their mutated photoprotective signal transduction under HL conditions. *DSR1*, the *UVR8* mutant, similarly expressed the same photoprotective genes as the *LHCSR1-Luc717* strain, irrespective of the mutated UV-inducible signal transduction. This interesting phenotype could be interpreted as compensational signal transduction via photosynthesis- and/or phototropin-dependent photoprotective signal transduction in this green alga^14^. Only *DSR29* exhibited LHCSR1-specific suppression (Fig. 4A and Supplementary Fig. S7), allowing the mutant to survive under HL-UV conditions, whereas all other mutants except *DSR1* showed obvious chlorophyll bleaching. Taking these results together, we could separate the mutants into “UV-specific” (*DSR1*), “*LHCSR1* promoter/transcript-specific” (*DSR29*) and “general photoprotective” (*CC4286*, *DSR10*, *DSR15* and *DSR28*) mutant groups (Fig. 5).

## Conclusion

In this study, we obtained various types of photoprotection mutants of *C*. *reinhardtii* and reconfirmed that *UVR8* is one of the genes responsible for UV-inducible *LHCSR1* gene expression. Since the UVR8 protein was recently investigated as a UV photoreceptor in *C*. *reinhardtii*, as it had been reported to function in land plants^11,21^, identification of the *UVR8* mutant indicated that our systematic screening utilizing UV irradiation and the LHCSR1-luciferase reporter system was reliable. It is plausible that the other single-locus insertional mutants (*DSR10* and *DSR15*) isolated in this work may indicate novel factors in photoprotection in green algae. In contrast to the *DSR1*, *DSR10* and *DSR15* mutants, we were unable to confirm single-locus *aph7* cassette insertion and/or mutation site(s) in the other mutants (*CC4286*, *DSR28* and *DSR29*), whose phenotype did not cosegregate with functional *aph7* insertion (i.e., hygromycin resistance). Since the *CC4286* and *DSR28* strains were obviously bleached under HL-UV conditions, it is plausible that the mutation(s) in these strains are critical for general photoprotection mechanisms in *C*. *reinhardtii*. To reveal the exact mutation sites of the mutants isolated in this study, further analysis including RESDA-PCR^22^ and/or whole-genome sequencing are required.

## Methods

### Strains and culture conditions

The WT *C*. *reinhardtii* strain *137c* (mt+ ) and the CC4286 strain were obtained from the Chlamydomonas Resource Center (http://www.chlamycollection.org). All strains and mutants were grown mixotrophically on TAP medium. Strains were grown under dim light (<10 μmol/m^2^/s) at 23 °C until mid-log phase (2–5 × 10^6^ cells/mL) for all experiments.

### Plasmid construction

To generate the LHCSR1-luciferase reporter gene, we constructed the plasmid pRT-GenD-LHCSR1-LUCnc as follows. An ~3.6 kb DNA fragment containing the *LHCSR1* gene with ~1.2 kb of its 5′-flanking and ~1.0 kb of its 3′-flanking regions was PCR-amplified from genomic DNA using the primer set *LHCSR1-Fw2-XhoI* (5′-TCACCTCGAGAGCATCCAACCCAGGAGCAG-3′)/*LHCSR1-Rv2-XbaI* (5′-GACTTCTAGAGGCGGCAGAGGTGTTGAACT-3′) (underlining denotes the restriction site created for subsequent cloning) and cloned into a XhoI/XbaI-restricted pRT-GenD-LHCSR3.1 plasmid^14^. The ClaI restriction site was introduced just before the stop codon of the *LHCSR1* gene by PCR-mediated site-directed mutagenesis, generating plasmid pRT-GenD-LHCSR1-ClaI. Then, a codon-optimized firefly luciferase gene fragment (*LUCnc*) was derived from the plasmid pCR2.1Topo-LUCnc^17^ by digestion with ClaI, and this fragment was cloned into the ClaI site of pRT-GenD-LHCSR1-ClaI, generating plasmid pRT-GenD-LHCSR1-LUCnc.

### Nuclear transformation and selection of *C*. *reinhardtii*

Nuclear transformations of *Chlamydomonas* were carried out by electroporation with a NEPA21 Super Electroporator (Nepa Gene, Japan)^23^. WT cells in the exponential phase (2 × 10^6^ cells/mL) were transformed with 300 ng of NotI-linearized pRT-GenD-LHCSR1-LUCnc plasmid. The transformed cells were selected under dim light at 23 °C on selective TAP plates containing 10 μg/mL paromomycin. *LHCSR1-Luc717* cells in the exponential phase (2 × 10^6^ cells/mL) were transformed with 20 ng of a PCR-amplified *aph7* cassette including the β-tubulin promoter and the *RBCS2* terminator. The transformants were selected on TAP plates containing 15 μg/mL hygromycin for later screening.

### Bioluminescence reporter assay

Mixotrophically grown transformants in 96-well microtiter plates were irradiated with low light (LL; white fluorescent light), UV light (fluorescent light with UV, generated using a T8 ReptiSun 10.0 UVB fluorescent bulb (Zoo Med Laboratories, CA)), or a monochromatic LED (blue, 450 nm; green, 530 nm; and red, 660 nm) at 30 μmol/m^2^/s. After 4 hours of light treatment, the transformants were mixed with luciferin (potassium salt; Promega, Madison, WI) at a final concentration of 100 μM and transferred into white 96-well microtiter plates (PerkinElmer, Norwalk, CT). Bioluminescence from the transformants was measured with a highly sensitive bioluminescent detector (model CL96; Churitsu Electric Corp., Japan).

### Linkage analysis of mutants

We first crossed the *LHCSR1-Luc717*(mt+ ) strain with the *CC4286*(mt−) strain. After measurement of UV-inducible bioluminescence in the progeny, we chose an mt− clone exhibiting a similar phenotype to the *LHCSR1-Luc717*(mt+ ) strain and crossed it with this strain. We chose one mt− clone exhibiting UV-inducible bioluminescence and named the clone *LHCSR1-Luc717*(mt−). For linkage analysis, mutants (mt+) were crossed with *LHCSR1-Luc717*(mt−). Then, mt+ and mt− cells were incubated on 1/2 N TAP (containing half as much nitrogen as TAP) plates for 4 days. The cells were suspended in M-N medium^24^ and incubated for 4 h with vigorous shaking under light to induce gamete formation. Then, mt+ and mt− gametes were mixed and incubated under light for 30 min to form zygotes, and the mixture was spread on a TAP plate. After 1 day of incubation under light, the plate was covered with aluminum foil and left for a week to allow the zygotes to mature. Tetrad analysis was performed with a stereomicroscope (Leica M80; Leica Microsystems, Bensheim, Germany). Progeny were obtained from the zygotes, recovered in 96-well plates containing TAP medium, and tested for hygromycin resistance and luciferase activity as described above.

### RT-PCR analysis

Total RNA from light-treated cells was extracted with the Maxwell RSC instrument (Promega) equipped with the Maxwell RSC simplyRNA Tissue Kit (Promega). The isolated RNA was quantified with the QuantiFluor RNA System (Promega), and the amounts of RNA were adjusted to 10 ng prior to reverse transcription. Reverse transcription was carried out with the ReverTra Ace qPCR RT kit (Toyobo, Japan) according to the manufacturer’s instructions. The obtained cDNA was diluted tenfold with distilled water (final conc. at 1 ng/μL) prior to PCR analysis carried out with KOD FX Neo DNA polymerase (Toyobo) on the SimpliAmp Thermal Cycler (Thermo Fisher Scientific, Waltham, MA). The number of PCR cycles used for each gene was 25 (*LHCSR1*), 24 (*LHCSR3*.*1*), 24 (*LHCSR3*.*2*), 26 (*PSBS1*), and 25 (*CBLP*). For quantitative RT-PCR, the G protein ß-subunit-like polypeptide (*CBLP*) gene was chosen as a housekeeping gene during light treatment. A real-time quantitative PCR assay was performed using the KOD SYBR® qPCR Mix (Toyobo) on the Light Cycler 96 system (Roche Diagnostics, Germany). The primers for PCR and qRT-PCR were *LHCSR1* (5′-TGTTGGCAGAATTGTGTGACATGG-3′ and 5′-GCCCATTTCTTATACATCCGATGCAC-3′), *LHCSR3*.*1* (5′-GCTTGTTCCCGCTCGAGC-3′ and 5′-GCTCCGTGGAGCCTGCTC-3′), *LHCSR3*.*2* (5′-CCGCTTGCTTCTGCTCAAGTTC-3′ and 5′-CTCTCGCCTGTTGTCACCATC-3′), *PSBS1* (5′-AGGGTAGAACAGCTATGGTTTCGT-3′ and 5′-CCGTCAGATCCCGTTCTCTCTG-3′), and *CBLP* (5′-GAGTCCAACTACGGCTACGCC-3′ and 5′-CTCGCCAATGGTGTACTTGCAC-3′).

### Genomic PCR analysis of the *UVR8* gene

PCR was performed on genomic DNA with the same DNA polymerase used for the PCR described above. The primers for the *UVR8* gene were *UVR8-Fw* (5′-ATGTACAATGGAGACCATCAGGAG-3′) and *UVR8-Rv* (5′-CGCGCCCGCTTACATGTCAC-3′), and those for the *aph7* cassette were *aph7-Fw* (5′-GTAAATGGAGGCGCTCGTTGATC-3′) and *aph7-Rv* (5′-CTCCCAGAATTCCTGGTCGTTC-3′).

### Immunoblot analysis

Immunoblot analysis was performed as described previously^14^. Antibodies against ATPB (AS05 085, rabbit polyclonal) and firefly luciferase (M095-3, mouse monoclonal) were obtained from Agrisera (Sweden) and MBL (Japan), respectively. Rabbit polyclonal antibodies against LHCSR1 and both the LHCSRs were raised and affinity-purified against the peptide SGKRTVSGKAGAPVP or LGLKPTDPEELK, respectively (Eurofins Genomics, Japan).

### High-light tolerance assay

Mutant cells in HS media^25^ were irradiated with either LL (white fluorescent light at 30 μmol/m^2^/s) or HL (including both UV fluorescent light and red monochromatic LED light (660 nm) at 300 μmol/m^2^/s) for 8 hours. Total RNA and protein extracts were obtained from cell cultures after 1 hour and 8 hours of light treatment, respectively. Chlorophyll fluorescence-based photosynthetic analysis was performed as follows. Maximum yields (Fm) were measured under dark conditions [after weak far-red (<5 μmol/m^2^/s) treatment for 30 min] using FluorCam 800MF (Photon System Instruments, Czech Republic). The maximum and steady-state fluorescence yields under light (Fm′ and F, respectively) were measured after actinic irradiation at 300 μmol/m^2^/s for 30 s. NPQ was estimated using the equation NPQ = (Fm − Fm′)/Fm′. The electron transfer rate (ETR) was estimated using the following equation ETR = (Fm′ − F)/Fm′ × 0.84 × 0.5 × light intensity.

## Supplementary information

Supplementary Figures and Tables
