# Supplementary material for: Isolation of photoprotective signal transduction mutants by systematic bioluminescence screening in Chlamydomonas reinhardtii
Source: Sci Rep. 2019 Feb 26;9:2820. doi: 10.1038/s41598-019-39785-z (PMC6391533; doi:10.1038/s41598-019-39785-z)
Supplement: Supplementary file 1 — Supplementary Figures and Tables [file 41598_2019_39785_MOESM1_ESM.docx]

**Isolation of photoprotective signal transduction mutants by systematic bioluminescence screening in *Chlamydomonas reinhardtii***

Ryutaro Tokutsu^1,2,3^, Konomi Fujimura-Kamada^1,3^, Tomohito Yamasaki^4^, Takuya Matsuo^5^, and Jun Minagawa^1,2,3^

^1^ Division of Environmental Photobiology, National Institute for Basic Biology, Nishigo-naka 38, Myodaiji, Okazaki 444-8585, Japan

^2^ Department of Basic Biology, School of Life Science, Graduate University for Advanced Studies, Okazaki 444-8585, Japan

^3^ Core Research for Evolutional Science and Technology, Japan Science and Technology Agency, Saitama 332-0012, Japan

^4^ Science and Technology Department, Natural Science Cluster, Kochi University, 2-5-1 Akebono-cho, Kochi 780-8520, Japan

^5^ Center for Gene Research, Nagoya University, Nagoya 464-8602, Japan

**Supplementary Information**

**Supplementary Figure S1. Bioluminescence activity in *LHCSR1-luciferase* reporter candidate strains.**

**
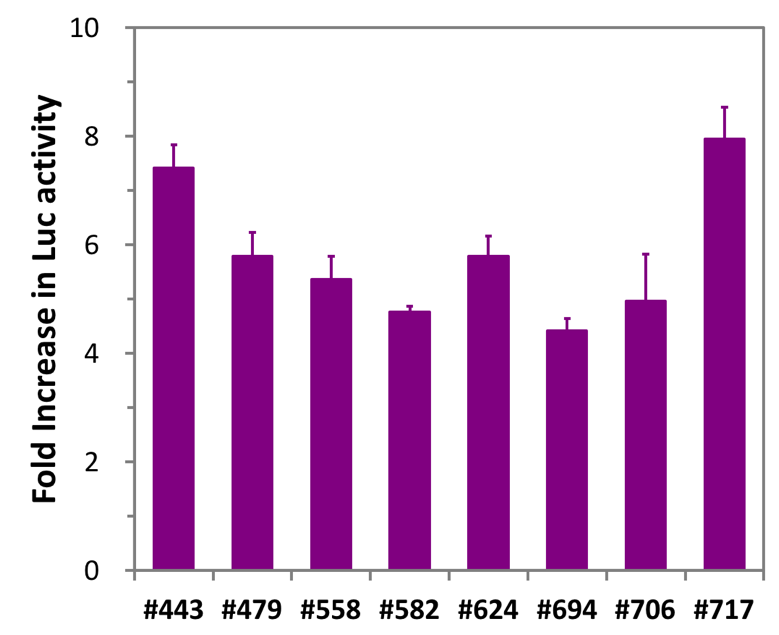
**Bioluminescence activity after 4 hours of UV illumination in the reporter candidate strains was normalized to the bioluminescence activity under LL. The intensities of the fluorescent light with UV was 30 μmol/m^2^/s. n = 3 biological replicates, mean ± s.e.

**Supplementary Figure S2. Immunoblot analysis of LHCSR1 and LHCSR1-luciferase fusion protein.**

Immunoblot analysis of LHCSR1 and LHCSR1-luciferase fusion protein in the reporter strains obtained in Fig. S1 using a specific antibody against LHCSR1. The images obtained from the same blot are shown. Short- (left) and long-exposure (right) blots were selected for visualizing LHCSR1 (~27 kDa) and LHCSR1-luciferase (~89 kDa) fusion proteins, respectively. Each label indicates the light treatment of cells as follows: D: dark, U: UV, B: blue (450 nm), G: green (530 nm), and R: red (660 nm). The intensities of the fluorescent light with UV and the blue (450 nm), green (530 nm) and red (660 nm) monochromatic LED lights were 30 μmol/m^2^/s.
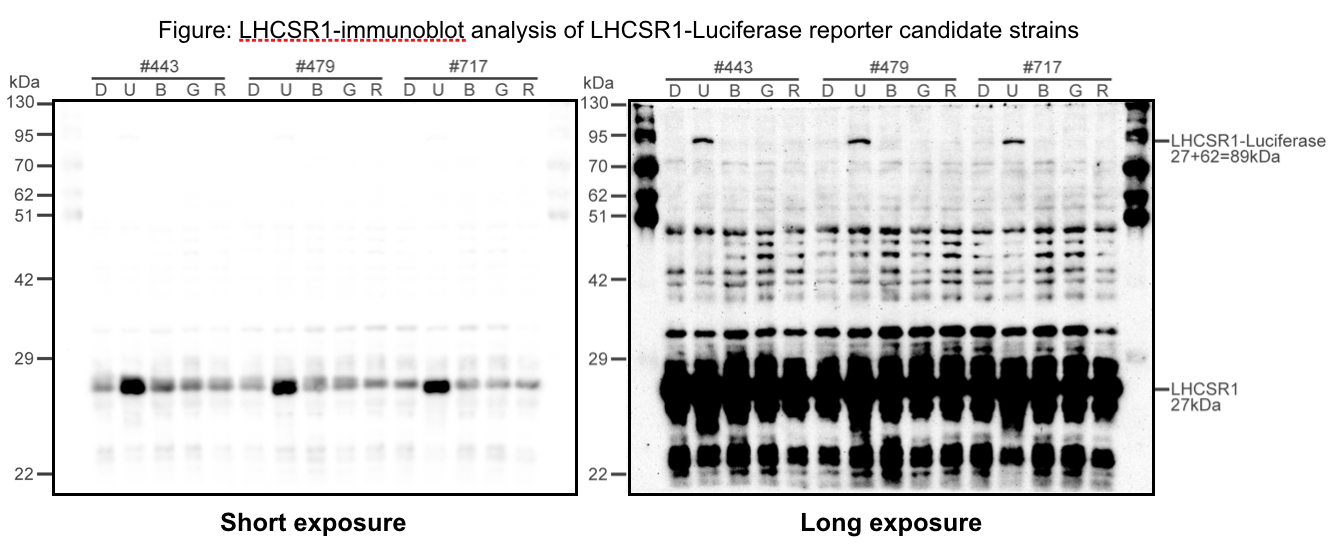


**Supplementary Figure S3. Immunoblot analysis of luciferase expression in the reporter strain.**

**
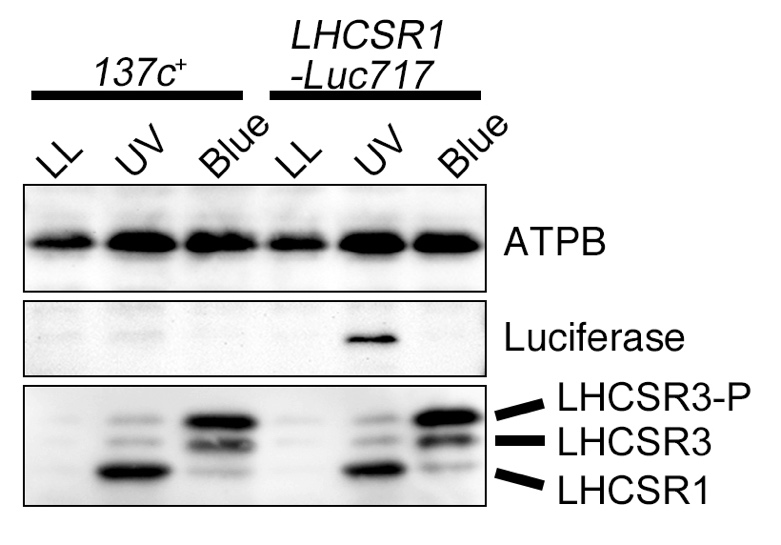
**Protein expression of luciferase and the LHCSRs after 4 hours of exposure of the wild-type and reporter strains to different light sources. The intensities of the fluorescent light with UV and the blue light (450 nm) were 30 and 100 μmol/m^2^/s, respectively. Immunoblot analysis was performed with specific antibodies against firefly luciferase and the LHCSRs [LHCSR1, LHCSR3, and LHCSR3-phosphorylated (LHCSR3-P)]. Samples shown here are representative of three biological replicates.

**Supplementary Figure S4. Nonphotochemical quenching of the reporter strain.**


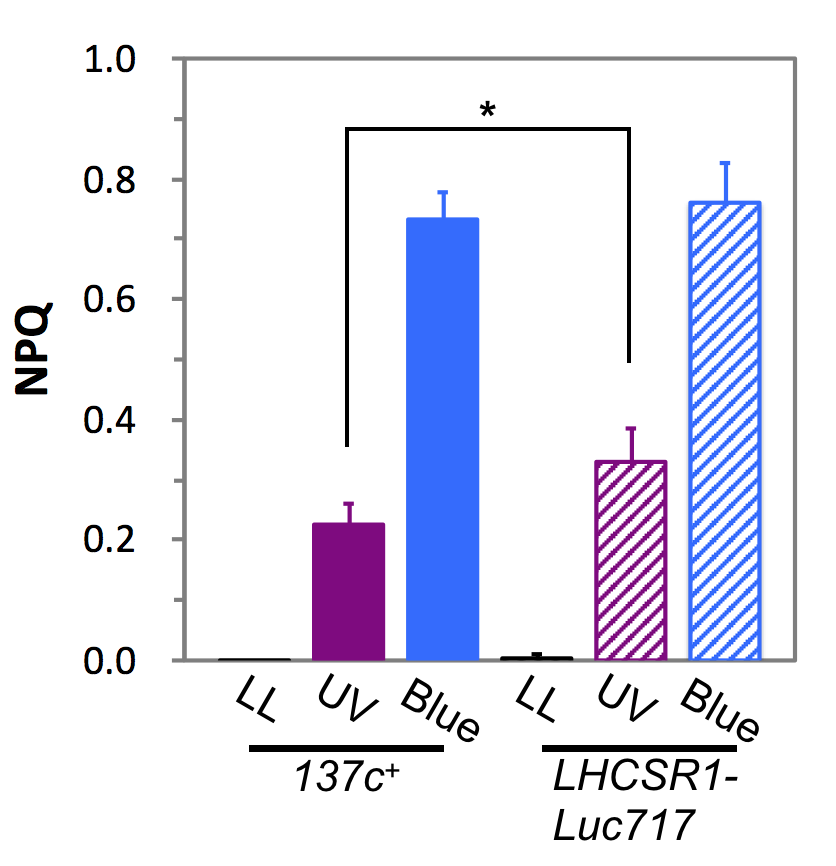
NPQ of cells (filled bar: 137c, and meshed bar: *LHCSR1-Luc717*) after 4 hours of exposure to light. The intensities of the fluorescent light with UV and the blue light (450 nm) were 30 and 100 μmol/m^2^/s, respectively. n = 3 biological replicates, mean ± s.e. Statistical significance was analyzed by Student’s t-test; * denotes P < 0.05.

**Supplementary Figure S5. *LHCSR1* gene expression in mutants.**


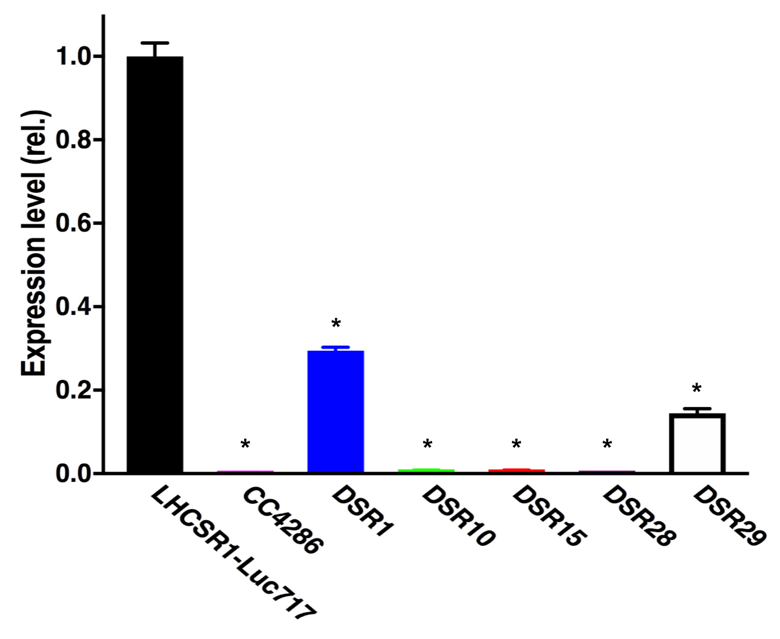
Quantitative gene expression analysis of *LHCSR1* by qRT-PCR. The *CBLP* gene was used as a housekeeping control gene. The colored bars indicate *LHCSR1-Luc717* (black), *CC4286* (magenta), *DSR1* (blue), *DSR10* (green), *DSR15* (red), *DSR28* (purple), and *DSR29* (white) expression levels. The gene expression levels of each mutant strain were normalized to those of the recipient strain (*LHCSR1-Luc717*). RNA samples were collected after an hour of UV irradiation. n = 3 biological replicates, mean ± s.e. The significance of values relative to the recipient strain value (*LHCSR1-Luc717*) was analyzed by Dunnett’s multiple comparison test; * denotes P Value as P < 0.05.

**Supplementary Figure S6. Photosynthetic electron transfer rate of mutants.**


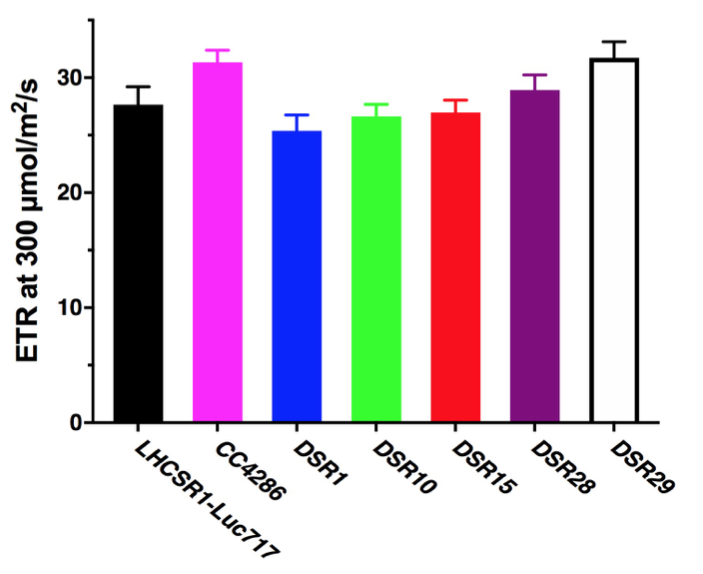
ETR of cells exposed to high-light conditions, as shown in Fig. 4. The colored bars indicate *LHCSR1-Luc717* (black), *CC4286* (magenta), *DSR1* (blue), *DSR10* (green), *DSR15* (red), *DSR28* (purple), and *DSR29* (white) ETRs. n = 12 biological replicates, mean ± s.e. No significant difference (at the level of P < 0.05) between mutant ETRs and the recipient strain (*LHCSR1-Luc717*) ETR was detected by Dunnett’s multiple comparison test.

**Supplementary Figure S7. Photoprotective gene expression of mutants.**


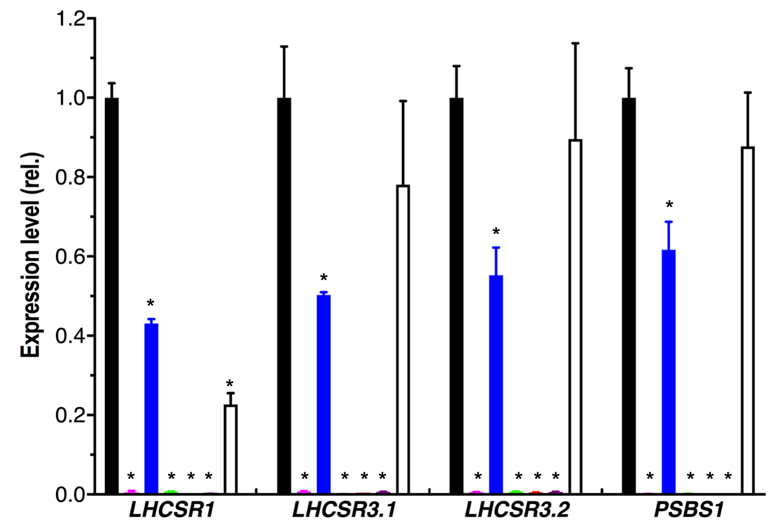
Quantitative gene expression analysis of *LHCSR1*, *LHCSR3.1*, *LHCSR3.2*, and *PSBS1* by qRT-PCR. The *CBLP* gene was used as a housekeeping control gene. The colored bars indicate the *LHCSR1-Luc717* (black), *CC4286* (magenta), *DSR1* (blue), *DSR10* (green), *DSR15* (red), *DSR28* (purple), and *DSR29* (white) expression levels. The gene expression levels of each mutant strain were normalized to those of the recipient strain (*LHCSR1-Luc717*). RNA samples were collected after an hour of irradiation under HL. n = 3 biological replicates, mean ± s.e. The significance of differences between mutant expression levels and the recipient strain (*LHCSR1-Luc717*) expression levels was analyzed by Dunnett’s multiple comparison test ; * denotes P Value as P < 0.05.

**Supplementary Table S1. Tetrad analysis of the mutants obtained in this study.**

| ***Mutant name*** | ***Number of***  ***tetrads tested*** | ***2:2 Segregation***  ***of hygromycin resistance*** | ***2:2 Segregation***  ***of luciferase activity*** | ***Number of***  ***cosegregated tetrads*** | ***Cosegregation***  ***rate (%)*** |
| --- | --- | --- | --- | --- | --- |
| *DSR1* | 12 | 12 | 12 | 12 | 100 |
| *DSR10* | 25 | 25 | 25 | 25 | 100 |
| *DSR15* | 25 | 25 | 25 | 25 | 100 |
| *DSR28* | 9 | 9 | 9 | 1 | 11.1 |
| *DSR29** | 2 | 0 | 2 | 0 | 0 |
| *CC4286* | 39 | - | 39 | - | - |

Progenies obtained during tetrad analysis were evaluated for both hygromycin sensitivity and luciferase activity. The number of tetrads and (co)segregation values are shown. The viability of *DSR29* progeny from single tetrads was too low; we therefore tested cosegregation of hygromycin resistance and luciferase activity in 25 surviving progenies as the random progeny of the *DSR29* mutant. Out of the 15 *DSR29* clones showing low luciferase activity, only six clones were hygromycin resistant, indicating that *aph7* cassette insertion was not responsible for the *DSR29* phenotype.
